# Seeing Through the Fog: The Ability to Resolve Ambiguity Reduces Dishonesty

**DOI:** 10.1177/01461672241305687

**Published:** 2025-01-07

**Authors:** Michael Puntiroli, Serhiy Kandul, Valéry Bezençon, Bruno Lanz

**Affiliations:** 1 University of Neuchâtel, Switzerland; 2 University of Zurich, Switzerland

**Keywords:** ambiguity, individual ability, disambiguate success, training, dishonesty

## Abstract

Ambiguity acts as a veil that can help conceal and justify dishonest behavior. While an individual’s ability to disambiguate information in a task may help remove the veil of ambiguity and thus promote honesty, the relationship between ambiguity, ability, and dishonesty is currently unexplored. To investigate this, we employed an experimental design where participants attempted to resolve an ambiguous task and reported their performance. Results showed that ambiguity and dishonesty increase in unison. Importantly, the participants who resolved ambiguity acted less dishonestly (Study 1). In Studies 2a, 2b, and 3, we increased participants’ ability by briefly training them to disambiguate the information presented in the task. The results showed that participants acted less dishonestly when their ability levels were increased. Overall, the findings indicate that dishonesty can be reduced not only by making tasks less ambiguous but also by enhancing an individual’s ability to successfully resolve ambiguity.

## Introduction

At the heart of most scandals, crimes, and acts of misconduct lies dishonesty. Ambiguous tasks are among the contexts that most favor dishonesty, with ambiguity relating to information that is open to interpretation and requires a degree of effort to be solved (Balcetis & Dunning, 2006; Schweitzer & Hsee, 2002). When faced with ambiguous tasks, people tend to use ambiguity as a justification to behave in self-serving ways, such as cheating and lying to gain an advantage (Pittarello et al., 2015; Shalvi et al., 2015). Using ambiguity to one’s own advantage is a widespread phenomenon, spanning most fields of human activity. In war, there is often the use of strategic ambiguity to gain an advantage over adversaries (Waltz, 1998). It can also be useful to avoid clarifying the motivations and drives of the enemy to one’s own people as a lack of understanding of the “other” can render brutal measures more palatable (Haslam, 2006). In diplomacy, diplomats can employ “constructive ambiguity,” leaving treaty terms or agreements purposefully vague to reap benefits at a later stage (Pillar, 1983). When applied to tasks at the workplace, whether it be working hours to fulfill, claiming back expenses, or borrowing company equipment, employees find it easier to justify acting in self-serving ways when tasks are ambiguous (Tenbrunsel & Messick, 2004). The main message is that ambiguity can give people an excuse to act in self-serving ways, resulting in dishonest behavior.

One way to decrease dishonesty is to remove ambiguity, which entails directly disambiguating the information in the task. However, disambiguation is not always feasible, as ambiguity may be naturally embedded into a task, or disambiguating procedures may prove too costly. Furthermore, most areas of human activity present some degree of ambiguity that leaves space for self-serving interpretations, and some have called this space “moral wiggle room” (Dana et al., 2007; Vu et al., 2023). In all these contexts, honest behavior may be encouraged by targeting the individual rather than disambiguating the information in the task. Previous research demonstrated that honesty can be promoted through moral training (Loe & Weeks, 2000), ethical training (Black et al., 2021), or by employing moral cues (Reynolds & Ceranic, 2007; Welsh & Ordóñez, 2014), although moral cues can backfire and even increase dishonesty (Zhao et al., 2019). In essence, these strategies aim to reduce dishonesty by appealing to a person’s honest side. However, it is currently unclear whether a reduction in dishonesty can be achieved without targeting honesty directly. More specifically, no literature to date exists on whether increasing a person’s ability to solve an ambiguous task decreases their proclivity to act dishonestly. Prior research shows that individuals with greater ability are more motivated to solve tasks (Ryan & Deci, 2000) and display more resilience (Masten, 2001), which should increase their chances of resolving the ambiguity in the task. When ambiguity in the task is reduced or eliminated by the individual, dishonesty should follow suit. What is currently unknown, and we seek to address in this research, are the ties between individual ability, intended as the ability to disambiguate information in a task, and dishonesty. We define dishonesty as the act of misrepresenting reality, for instance, by providing inaccurate information, whether intentionally or unintentionally, that results in a direct personal benefit. Crucially, many instances of everyday life require disambiguation to be able to act truthfully. Within the context of investigative journalism, a diligent journalist must first attempt to resolve ambiguity surrounding a case by investigating their leads, then decide if to report a true story or to peddle a sensational story. An academic researcher must first see through the fog of the data to investigate a research question, then report results that may be accurate or doctored. At work, a person can receive an email written in highly technical language that requires some effort to disambiguate, then react to the content of that email. Of course, some people will put effort into attempting to disambiguate the task at hand, while others will not. Some of those who attempt to disambiguate the task will succeed at fully uncovering the facts, and removing the veil of ambiguity, while others will fail. We are interested in determining whether enabling people to successfully disambiguate the task discourages dishonest behavior. We therefore ask the following research question: Does enhancing an individual’s ability to successfully disambiguate a task decrease dishonesty?

The remainder of the article is structured as follows. We first provide an overview of the literature linking ambiguity to dishonest behavior. Then, we examine the literature indirectly linking ability levels to honesty and develop our conceptual model and hypotheses. One pre-study and four main studies were conducted to test those hypotheses. We end with a general discussion with theoretical and practical implications.

## The Link Between Ambiguity and Dishonesty

There are usually external costs to acting dishonestly, such as punishments and penalties (Becker, 1968; Grolleau et al., 2016; Thielmann & Hilbig, 2018). However, the costs are also internal, with the erosion of one’s own positive self-image (Abeler et al., 2014; Battigalli et al., 2013; Pyszczynski et al., 2004; Rosenbaum et al., 2014). This explains why people cheat more when their actions are more easily justifiable (Bassarak et al., 2017; Klein et al., 2017; Pittarello et al., 2015; Shalvi et al., 2011), as the availability of justifications, to others or oneself, reduces the internal cost of dishonesty. Ambiguity can be thought of as a gray area, or as fog, that offers the opportunity for people to easily conjure up justifications. Taxation procedures (Alm et al., 2010), symbols (Balcetis & Dunning, 2006), data (Kale et al., 2019), and distances (Pittarello et al., 2019) can all be ambiguous. Across these contexts, ambiguity offers a certain degree of freedom, where people can act dishonestly and still look moral to themselves and others (Shalvi et al., 2012). Crucially, what drives dishonesty is predominantly the ease with which the facts can be distorted, rather than the size of the gains that can be obtained (Hilbig & Hessler, 2012). This whole body of research on ambiguity suggests that minimizing justifications for dishonesty will minimize dishonesty.

Finally, ambiguity in tasks is sometimes unresolvable, as was the task designed by Balcetis and Dunning (2006), where participants cannot perfectly disambiguate the presented information. Other times ambiguity can be resolved, as in the case of estimating distances from a cross to a target (e.g., Pittarello et al., 2015), or taking time to understand taxation procedures (Alm et al., 2010). In these situations, there is scope for an individual’s ability to play a crucial role in successfully disambiguating the information. It is this resolvable ambiguity that we focus on.

## The Role of Ability on Dishonesty

Previous research indirectly linking ability levels to moral behavior shows that increased ability encourages people to take control, as opposed to disengaging from a task or situation (Bandura, 1991, 1997). When people feel they are fully able to perform their activities, they are more intrinsically motivated and exhibit higher levels of well-being (Ryan & Deci, 2000). This line of reasoning suggests that increased ability may put people in a better position to resolve ambiguity present in the task. In support of this prediction, Pascual-Ezama et al. (2020) show that participants who simply skip rolling a die, when instead they were supposed to, go on to cheat the most. Similarly, Gross and De Dreu (2021) also found that participants who do not follow task rules go on to cheat more when given the chance. Pascual-Ezama et al. (2020) speculated that when people do not discover the “truth,” as was the case in their study when participants skipped rolling a die, this gives them more leeway to act dishonestly. By contrast, those most able to resolve ambiguity should find it easier to uncover the truth, and uncovering the truth is expected to discourage dishonest behavior. Furthermore, research suggests that as individuals become more able in a particular domain, they tend to process information more quickly and efficiently (Ericsson & Lehmann, 1996), increasing the chances that such individuals successfully resolve an ambiguous task. Once the ambiguity is resolved, the self-justification hypothesis posits that it should be more difficult to act dishonestly, given that the moral wiggle room has been removed.

An intervention that facilitates the disambiguation of an ambiguous task should silence self-serving justifications that ambiguity promotes and thus encourage people to act less dishonestly. One approach to achieve this may be to increase one’s abilities through training. People who have been successfully trained on a task, with their ability levels objectively increased, are expected to find an ambiguous task easier to resolve. In other words, training may reduce the level of effort required to solve the task, reducing the scope for self-serving justifications. For example, if individuals successfully complete a task several times during training sessions, they cannot argue that they lack understanding of what the task entails, to justify cheating to themselves. Greater ability provides the individual with a set of benefits that allows them to better cope with stressful experiences, preventing an avoidant approach toward these experiences (Bandura, 1977). Greater ability does not only aid the individual in taking control when adversity arises, but it also changes the way the individual perceives the external environment (Bandura, 1977). We therefore propose that increasing ability levels should make it more difficult for individuals to produce self-serving justifications. In sum, people with a greater ability to disambiguate information in a task should resolve ambiguity at a higher rate. Once ambiguity has been solved, the likelihood of acting dishonestly should be reduced, as self-justifications for dishonest behavior become more difficult to conjure up.

## The Current Investigation

**Figure 1.**
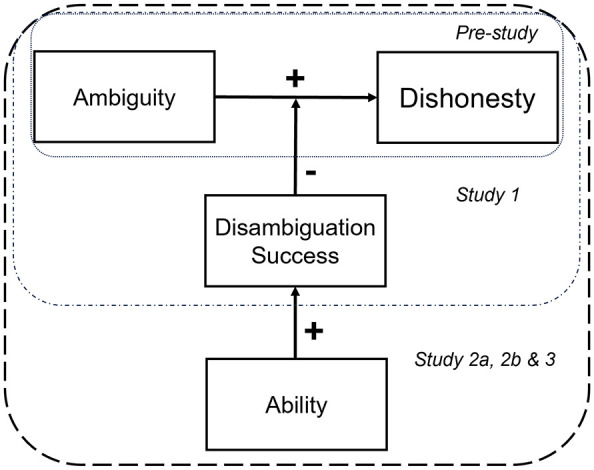
Illustration of the Experimental Framework and the Sequence of Studies. *Note. Ability* equates to a person’s ability to solve ambiguity in a task, and *Disambiguation Success* equates to the rate at which ambiguity in the task is resolved.

We are interested in reducing dishonesty levels in contexts where people have time to disambiguate the task, as might occur when filling out a questionnaire or interpreting an email in a foreign language. We theorize that people trained at resolving ambiguity in a task will be less likely to act dishonestly than untrained people because they will be better at disambiguating the task, making it more difficult to justify dishonest behavior (Figure 1). For that reason, increasing an individual’s ability to disambiguate the information present in the task may have the same effect as reducing the ambiguity that is present in a task.

We predict that ambiguity leads to more dishonest behavior (H1, Study 1), thus replicating prior findings with a novel procedure. Then we predict that greater disambiguation success will decrease dishonesty in ambiguous tasks (H2, Study 1). Crucially, we expect that greater ability decreases dishonesty in ambiguous tasks by increasing the chances of disambiguation success (H3, Study 2a, 2b, and 3). Study 2a and 2b test H3 with two different experimental paradigms, using a two-step procedure (first disambiguation, then behavior). Study 3 tests H3 using a one-step procedure, combining disambiguation and behavior all in one stage, allowing participants the option to skip the disambiguation phase.

## Study 1: Ambiguity and Dishonesty

### Method and Participants

Our participants were recruited from the online panel provider *prolific.co.uk*, matching the criteria of being older than 18 years, located in the United Kingdom, and using English as a first language. Using the statistical program G*Power 3.1 (Faul et al., 2009), we estimated that a minimum sample size of 470 was necessary given our predicted medium-sized effect (f2 = 0.20), given α = 0.05, using two independent groups. We decided to test a total of 600 participants (*M*_Age_ = 37; 36% males), which reduced to 585, with 15 failing one of two trivial attention checks. The first attention check question was positioned at the beginning of the study, “World War II comes after World War I” measured from 1, strongly disagree to 7 strongly agree, while the second appeared toward the end, “The main task consisted of. . .” followed by three options: one correct sentence about the study and two entirely implausible answers). Double the sample size was used in the ambiguity condition to analyze effects pertaining to effort and disambiguation success, clearly only possible in that condition. All participants were paid for their efforts according to U.K.’s minimum wage.

In keeping with the journal’s guidelines, we report all manipulations, measures, and exclusions in this and the following studies. All data files, for this and the following studies, can be found at: https://osf.io/5xuzt/?view_only=cc3d41872d114f9f86135552ea1ef359

### Materials and Procedures

**Figure 2.**
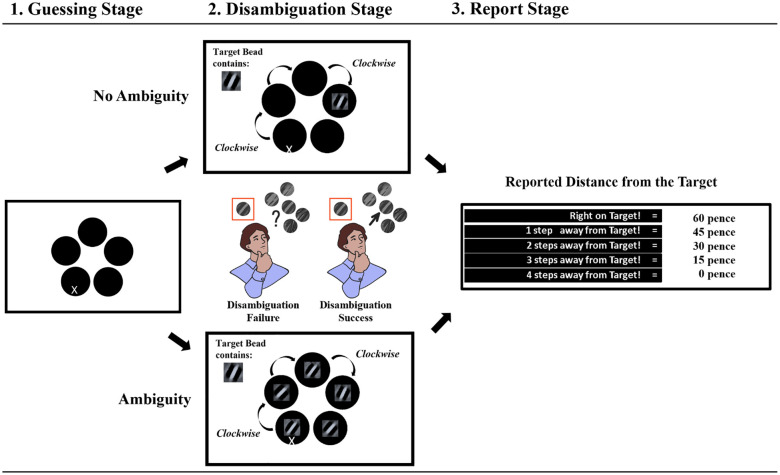
The Experimental Sequence Presented to Participants. *Note.* Participants were first asked to guess the location of the target bead and to remember their choice (Guessing Stage). In this example, the participant guessed the bottom left bead, indicated with a white cross. Then participants attempted to disambiguate the image (Disambiguation Stage) by trying to identify the target bead (randomly selected by computer) in one of two possible ambiguity conditions, No Ambiguity or Ambiguity, depending on the presence of distractors patches. The Disambiguation Stage could either be successful, meaning the correct target bead was identified, or it could be unsuccessful. The participants then counted the distance from their chosen bead to the target bead (in the clockwise direction). In this example, the target bead was to the right, in the 3 o’clock position in both conditions and was therefore three steps away from the chosen bead with the white cross. Finally, participants reported how close they were to the target bead, that is, the distance (Report Stage).

The experiment consisted of three main stages (see, Figure 2). In Stage 1, the guessing stage, participants were first presented with a “necklace” of five beads and asked to guess which bead would be randomly chosen by the computer. Participants were simply told to “Please choose one bead and Remember it Now—You have 1 shot at guessing where the Target will appear” followed by “You don’t have to click on it, just be sure you remember which one you picked.” This meant that participants never revealed their guesses to the experimenter and therefore operated under complete unobservability.

In Stage 2, the disambiguation stage, one of the five beads was randomly chosen by the computer to be the target and then revealed to participants as the only bead that contained a specific pattern of a Gabor patch, a popular stimulus used in experimental psychology (e.g., Rolfs et al., 2011; Szinte et al., 2019). In line with similar studies requiring that participants disambiguate either a target image (e.g., Balcetis & Dunning, 2006) or the position of a target in relation to other stimuli (e.g., Pittarello et al., 2015), we ask participants to disambiguate a target stimulus from a number of distractors. The target was either presented unequivocally, where it was immediately clear which bead was the target (No Ambiguity Condition), or it was presented among similar-looking beads, that is, distractors, making it difficult to identify the target bead (Ambiguity Condition). We therefore employed a between-subjects design, randomly allocating participants to one of the two ambiguity conditions. Participants were then asked to identify the target by clicking on the respective bead on the screen before moving to the next stage.

Finally, in Stage 3, the reporting stage, participants were asked to report how close their chosen bead was to the target. They read «Please count the steps from your bead to the target» followed by «You will be paid according to the following bonus scheme». The incentive scheme instructed them that the closer they were to the target the higher their bonus payment would be. Therefore, there was a financial incentive to be as close as possible to the target. The reported distance was a scale from 0 to 4 and acted as our measure of “dishonesty,” where a smaller distance implies more dishonesty. After seeing the incentive scheme, participants had to compute, and report, the distance from their guessed bead to the target bead in the clockwise direction. They had been instructed how to do so beforehand, with a series of examples. Importantly, as it was not possible to know which bead was picked by a participant during the guessing stage, that is, Stage 1, there was no possibility to verify whether each individual participant was telling the truth. This is a typical feature of designs in the dishonesty literature, allowing participants maximal freedom to behave as they see fit. In line with our definition of dishonesty, participants could intentionally lie, inflating their performance to reap financial benefits, or could make self-serving mistakes of a largely unintentional kind (e.g., Heyman et al., 2020; Pittarello et al., 2015). Both forms of misreporting that directly benefit the individual are labeled «dishonesty».

While dishonesty could not be observed at the individual level within this experimental design, it could instead be observed at the aggregate level, per condition. Given the randomization of the target bead, we knew that fully honest reports would produce a theoretical average report of 2.0 (two steps away from the target). This average report was also observed in unincentivized pre-tests, which empirically confirmed the theoretical benchmark for honesty. Therefore, average reports below 2.0 would indicate that participants were «decreasing» their true distance to the target bead. A preliminary study validated our method and confirmed that participants act more dishonestly in the presence of greater ambiguity (see pre-study in Appendix A). Overall, this experimental design allowed us to manipulate ambiguity by rendering the patch at the target bead location more or less similar to the distractor patches. This study’s design and hypotheses were preregistered (https://osf.io/jud5e/?view_only=fc2210a34ea449fc9b7817c22de8efd3).

In summary, the task asked that participants do the following: (a) attempt to disambiguate the information in the task, that is, identify the target among distractors and (b) report their performance, that is, how close their chosen bead was to the target. Data were analyzed using RStudio version 4.0.1 (R Core Team, 2020) and SPSS, version 28.

### Results

#### Main Effect of Ambiguity on Dishonesty

We began by verifying whether ambiguity led to more dishonesty. People in the ambiguity condition reported significantly shorter distances to the target, meaning more dishonesty (*M* = 1.02; *SD* = 1.33), than those in the no ambiguity condition (*M* = 1.66; *SD* = 1.44), *t*(377)=5.22, *p* <.001, confidence interval [CI]: [.40, .88.], Cohen’s *d*=0.46, supporting H1.

#### Effect of Disambiguation Success on Dishonesty

Next, we ran a linear regression model, assessing the effect of “disambiguation success” on dishonesty, within the Ambiguity condition, controlling for individual effort, measured as the time spent on the task. “Disambiguation success” was a significant predictor in the model, *b* = .34, *t*(379) = 2.49, *p* = .013, 95% CI [.07; .62], indicating that those who successfully resolved ambiguity acted less dishonestly.

The results indicate that disambiguation success decreases dishonesty (confirming H2). Next, we turn to examine whether greater ability levels increase the rate of disambiguation success and impact the proclivity for dishonesty.

## Study 2a: Increasing Ability Through Training

While Study 1 indicated that resolving ambiguity decreases the likelihood of acting dishonestly, Study 2 will examine whether increasing ability levels through a training session decreases dishonesty levels in an ambiguous task by increasing the chances that people successfully resolve ambiguity. This study’s design and hypotheses were preregistered (https://osf.io/zxwpe/?view_only=fc2210a34ea449fc9b7817c22de8efd3).

### Method and Participants

We randomly assigned participants to one of three conditions: a “control” condition (no ambiguity), a “control training” condition, and a “ability training” condition. The control training condition aimed at familiarizing participants with the task without improving their ability to disambiguate the task. By contrast, the ability training condition aimed at improving one’s ability to disambiguate the task. For a medium effect size (f2= 0.15, given α = 0.05) utilizing a mediation model, Fritz and MacKinnon (2007) suggest that the required sample size may vary from *N* = 71 to N = 391. Given that one of our analyses involves a multi-categorical independent variable, a sample size of 600 should ensure that our study is well-powered to detect medium-sized effects. Therefore, we recruited 600 participants on *prolific.co.uk* (*M*_Age_ = 35; 31% males) and a simple attention check failed by 12 participants led to a final sample of 588 participants.

### Materials and Procedure

We followed the same general procedure as in Study 2. The “control” condition (*N*=197) with no ambiguity remained the same as the previous experiments, while the “control training” condition (*N*=200) and the “ability training” condition (*N*=191) employed the High ambiguity condition. The “control training” session consisted of six practice rounds where participants clicked on the target bead, which was the only bead containing a Gabor patch, and then progressed to the next round. This rather trivial training session simply familiarized participants with the task.

In the “ability training” condition participants also carried out six practice rounds where they had to identify the target bead containing the displayed Gabor patch, while all non-target beads contained distractor Gabor patches of similar tilt. Once the target was identified, participants clicked on it. If the selected target was correct, the participant progressed to the next training round, if not, they would continue to select beads until they selected the correct one.

### Results

#### Main Effect of Ambiguity and Training on Dishonesty

**Figure 3.**
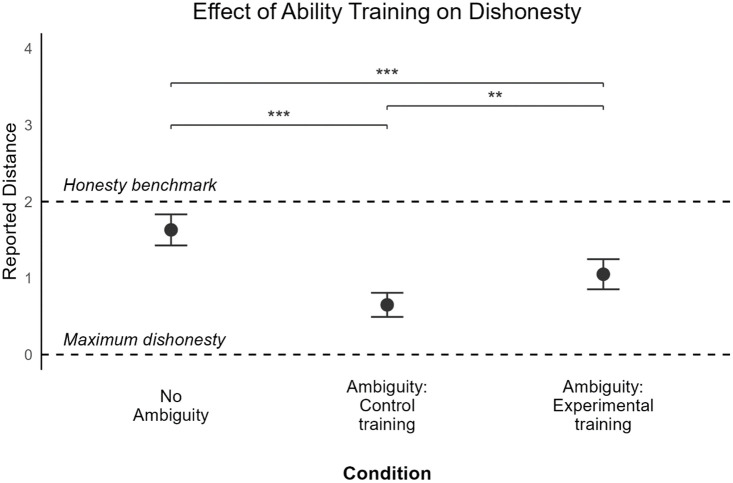
Illustration of the Reported Distance From Chosen Bead to the Target Bead, Averaged Per Condition. *Note.* Lower reported distances indicate more dishonesty. The dashed line on y-axis represents a theoretical benchmark of complete honesty. Error bars represent the standard error of the mean (+-2). *p*<.05 (*); *p*<.01 (**). The No Ambiguity condition acts as the control condition. The Ambiguity conditions act as the treatment conditions where higher dishonesty is expected in the Control Training condition compared with the No Ambiguity and the Experimental Training conditions.

To test for dishonesty differences between the experimental conditions, we employed a one-way between-subjects analysis of variance (ANOVA). Results highlighted a statistically significant difference in the reported distance between the conditions, *F*(2,585) = 27.95, *p* < .001 (Figure 3). Planned comparisons showed that the conditions differed significantly from each other, with the control condition leading to larger reported distances, *M* = 1.62; *SD* = 1.42, compared with the ambiguity condition with control training, *M* = 0.65; *SD* = 1.12, *p* < .001; CI: [.07, 1.22], Cohen’s *d* = 0.76 and with ability training, *M* = 1.06; *SD* = 1.35, *p* < .001; CI: [.03, .81], Cohen’s *d* =0.4.

A significant difference in the reported distance also emerged between control training and ability training conditions, *p* = .002; CI: [−.67, −.16], Cohen’s *d*=0.33. These results confirmed once again that the presence of ambiguity leads to greater dishonesty, in line with H1, but also that increased ability levels help decrease dishonesty when ambiguity is present, supporting H3. In fact, despite both training conditions presenting participants with the same level of ambiguity (i.e., high ambiguity), only those who had their ability levels increased acted less dishonestly on average.

#### Mediating Effect of Disambiguation Success on Dishonesty

Next, we aim to see whether those in the ability training condition acted less dishonestly than those in the control training condition because of their increased disambiguation success, supporting the notion that increased ability helps people see through the fog of ambiguity. First, we ran a linear regression measuring the effect of disambiguation success on reported distance. The overall model was significant, *F*(2, 390) = 19.62, *p* < .001, *R*^2^ = .09, with statistically significant positive effects of “disambiguation success,” *b* = .53, *t*(390) = 4.28, *p* < .001, 95% CI [.29; .77]. These results were consistent with the results of Study 1, indicating that increased disambiguation success reduced dishonesty levels (supporting H2).

To test the effects of increasing ability levels on dishonesty, we ran a multi-categorical mediation analysis using the MeMoBootR R package (Buchanan, 2018), entering the three experimental conditions as independent variables. A mediation analysis with bootstrap samples was preferred over alternatives because our primary interest was in assessing the specific relationships between the variables, rather than promoting a global model. The control condition was selected as the reference condition. Reported distance was the dependent variable, while disambiguation success was the mediator. The full pattern of results is illustrated in Appendix B. Relative to the control condition, the average reported distance was lower in the control training and ability training conditions (direct effects), indicating greater dishonesty in these two training conditions. This is unsurprising, as ambiguity was present only in the training conditions and not in the control condition. Both control training and ability training presented ambiguity and led to significantly less disambiguation success than the control condition with no ambiguity: respectively, *b* = −2.15, *p* <.001, *b* = −1.57, *p* < .001. Note that the strength of the effect reflects the comparison with the control condition, and therefore significant negative coefficients indicate the size of the “advantage” of the control condition. In line with our prediction, the control training led to significantly less disambiguation success compared with the control, than did the ability training compared with the control. Disambiguation success significantly predicted reported distance, *b* = .22, *p* < .001, confirming once again H2. We conclude that the effect of ambiguity and of the training is partially mediated by one’s ability to solve ambiguity.

For further insights, we examined how disambiguation success and dishonesty are shaped by individual effort, as in the time spent attempting to resolve ambiguity. It has been previously suggested that those who put no effort into tasks, and simply skip them, act the most dishonestly (Pascual-Ezama et al., 2020). And, in line with this, one would imagine that more effort spent disambiguating the task would lead to less dishonesty. These additional insights centered upon effort, disambiguation success, and dishonesty levels can be seen in Appendix C.

Furthermore, we checked for the effect of gender and saw no effect on any of the results reported. Specifically, we measured gender as *male* (1), *female* (2), or *other* (3), and we created dummy variables for female and other, while male was the reference condition. These dummy variables neither predicted disambiguation success nor reported distance nor did they alter the results presented above when used as control variables.

## Study 2b: Replicating the Effect of Training on Dishonesty

This study aimed at replicating the effects of Study 2a using a different experimental design and a different form of ambiguity. Specifically, we sought to once again observe the positive effect of ambiguity on dishonesty (H1) and of ability training on disambiguation success, leading to less dishonesty in ambiguous tasks (H2, H3). We therefore aim to add external validity to the results obtained in Study 2a.

### Method and Participants

A between-subjects design was employed where participants were randomly assigned to one of three conditions: a “no ambiguity control condition” (Condition 1), an “ambiguity condition with control training” (Condition 2) containing a training session aimed at familiarizing participants with the task but not aimed at increasing their ability to disambiguate the information in the task, and an “ambiguity condition with experimental training” (Condition 3) containing a training session aimed at increasing participants’ ability to disambiguate the information in the task. A total of 600 participants were recruited from prolific.co.uk (*M*Age = 37; 46% males) and a simple attention check failed by 18 participants led to a final sample of 582 participants.

### Materials and Procedure

**Figure 4.**
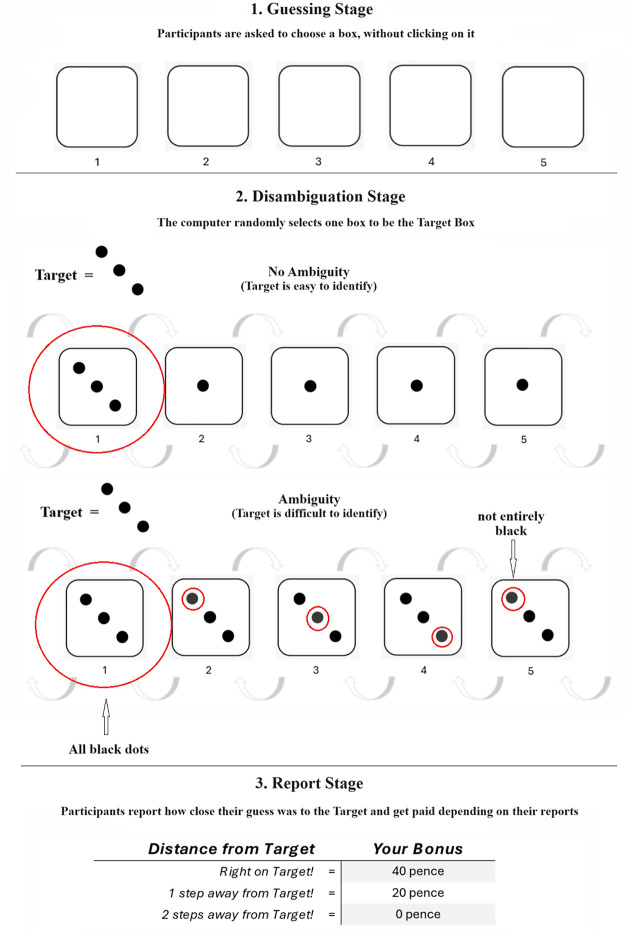
Experimental Sequence Presented to Participants in the Experiment. *Note.* Guessing Stage, followed by a disambiguation and the report stage. The experimental conditions differ in the disambiguation stage: In the control condition without ambiguity (Condition 1), the target box (box #1 in this example) is immediately identifiable as the only box containing three black dots. In the ambiguity conditions (Conditions 2 and 3), the target box (box #1 in this example), is presented among non-target boxes that greatly resemble the target box. These non-target boxes contained two black dots (RGB 0,0,0), and one dark gray dot (RGB 56,56,56) indicated by red circles). The circles in red were not shown to participants, that is, they had to disambiguate the task by identifying the target box.

Similar to previous studies, participants first received task instructions, informing them that they would be presented with five boxes and that their goal was to guess the box that would be selected by the computer. The initial examples informed the participants that the target box was always the box containing the three black dots. The target box was presented with or without ambiguity. In the no ambiguity condition, the non-target boxes lined next to the target box contained one black dot in the middle of the box (condition 1, *N*=195). This made the target box immediately identifiable as the only box containing three black dots. In the ambiguity conditions, the distractor boxes also contained three dots, two of which were black, while one was dark gray. We calibrated the darkness of the dark gray dot on the distractors, to make the task ambiguous but not impossible to solve. This was achieved by using a dark gray whose RGB values were 56, 56, and 56. Participants in the ambiguity conditions either took part in a “control training” condition (condition 2, *N*=198) or in an “experimental training” condition (condition 3, N=189). Those in the “control training” condition familiarized themselves with the task instructions and procedure over five trials. In each trial, the goal was to identify the target box without ambiguity being present, which meant that the non-target boxes were not distracting, as they only contained one central black dot. Those in the “experimental training” condition instead were required to identify the target box with ambiguity present. This meant completing five training trials where the target box was presented along with distracting non-target boxes that greatly resembled the target box. The rationale was to train participants to become efficient at disambiguating the target from the non-target boxes in the experimental training condition while leaving their skills unaltered if they took part in the control training condition. In both training conditions, participants could only progress to the next trial once they correctly identified the target box. In the main experimental trial, participants first made their guess, then attempted to disambiguate the information in the task by clicking on the box they thought was the target, followed by the report stage where they stated how far their chosen box was from the target. Figure 4 summarizes the flow of the experiment.

Following the instructions, and the training phase for participants in the two ambiguity conditions, came the main task. Similarly to the previous design, this consisted of a guessing stage (Stage 1) requiring participants to choose one of five boxes presented on screen and to remember which box they chose. The boxes were numbered, helping participants to better select and remember their boxes. Importantly, participants’ guesses were never disclosed to the experimenter who had no way of knowing which box was chosen. What followed was the disambiguation stage (Stage 2), where the computer selected a target box by revealing one box containing three diagonally placed black dots. The target box was either revealed unequivocally («no ambiguity condition») or alongside distracting non-target boxes rendering its identification ambiguous («control training» and «ability training» conditions). Participants were asked to identify the target box by clicking on it, providing us with a measure of disambiguation success, coded=1, if the target box was identified correctly, and 0 if otherwise.

Next, participants progressed to the report stage (Stage 3), where they reported the distance from their chosen box, in Stage 1, to the box they identified as the target box, in Stage 2. Participants computed the shortest distance from their chosen box to the target box and to do so they could count the steps in any direction (see Figure 4). The reported distance was our main dependent variable, acting as a measure of dishonesty. There were three possible values of reported distance: right on target (coded =1), one step away (coded=2), and two steps away (coded=3). Given the random selection of the target box by the computer, fully honest reports per condition produce an average report of 2.0 («one step away»), acting as our honesty benchmark. Deviations from the honesty benchmark toward 1.0 («right on target») indicated dishonesty, in the form of «inflated» performance. Reports were incentivized in the following way: Participants got an extra bonus of 40 cents for «right on target», 20 cents for «one step away» and 0 cent when they reported being «two steps away». There was therefore an incentive to report shorter distances to the target.

### Results

#### Main Effect of Ambiguity and Training on Dishonesty

A one-way between-subjects ANOVA aimed to test for possible differences between conditions in the reported distance from one’s chosen box to the target. The results highlighted a statistically significant difference in reported distance between the conditions, *F*(2,582) = 11.56, *p* < .001, with planned pairwise comparisons showing that reported distances in the control condition (*M* = 1.74; *SD* = 0.76) were significantly larger than in the ambiguity condition with control training, M = 1.40; *SD* = 0.67, *p* < .001; Cohen’s *d* = 0.48. Therefore, we replicated the main effect of ambiguity, where ambiguity leads to more dishonesty (supporting H1). Furthermore, the average reported distance in the ability training condition, *M* = 1.61; *SD* = 0.72, was significantly higher than in the control training *p* =.004, Cohen’s *d* =0.31 and only marginally different from the average report in the «no ambiguity» condition: *p* =.069. Both the ability training and the control training conditions presented the participants with the same ambiguous task, with the sole difference between conditions being the type of training session that preceded the main task. The results indicate once more that training reduced dishonesty in ambiguous tasks (supporting H3).

#### The Mediating Effect of Disambiguation Success on Dishonesty

First, we analyzed whether the conditions produced different disambiguation success scores. We employed a one-way between-subjects ANOVA. The results indicated significant differences in disambiguation success: *F*(2, 580)=81.4, *p* <.001. Planned comparisons revealed that disambiguation success in the no ambiguity condition (95.4%) was significantly higher than in the ambiguity conditions (*p* < .001). Disambiguation success in the experimental training (72.6%) was significantly higher than in the control training condition (43.4%), *p* < .001. Overall, the ability training session proved effective at increasing disambiguation success.

Next, we again verified whether increased disambiguation success on the part of those who underwent the ability training was the explanation for their reduced dishonesty. We ran a linear regression to measure the effect of disambiguation success on reported distance. The overall model was significant, *F*(1, 580) = 26.51, *p* < .001, *R*^2^ = .04, with a statistically significant positive effect of “disambiguation success,” *b* = .33, *t*(580) = 5.15, *p* < .001, 95% CI [0.21, 0.46]. These results are consistent with the results of Studies 1 and 2a, indicating that increased disambiguation success reduces dishonesty levels (supporting H2).

Then, we ran a multi-categorical mediation analysis as performed in Study 2a, entering the conditions as the independent variable and employing the control condition as the reference condition. Reported distance was the dependent variable, while disambiguation success was the mediator. The reported distance was lower in the “ambiguity + control training” and “ambiguity + ability training” conditions compared with the reference condition with no ambiguity, that is, total effect, indicating greater dishonesty in these two training conditions (respectively *b* = −0.34, *p* <.001; *b* = -0.13, *p* =.07). This is expected since both control training and ability training conditions presented ambiguity and led to significantly less disambiguation success than in the control condition with no ambiguity: respectively *b* = −0.52, *p* <.001, *b* = −0.22, *p* <.001. The mediator “disambiguation success” significantly predicted reported distance, *b* = .24, *p* < .001, confirming once again H2. The indirect effects of the ambiguity conditions on reported distance (via disambiguation success) constituted 0.37 and 0.42 of the total effects for Condition 2 (“Ambiguity + control training”) and Condition 3 (“Ambiguity + ability training”), respectively.

## Study 3: Effectiveness of Training Without “Forced” Disambiguation

Studies 2a and 2b indicated that increasing ability levels through an ability training session decreases dishonesty levels in an ambiguous task when participants are required to attempt to solve ambiguity in the task. In other words, the study designs so far have employed an explicit disambiguation stage where participants attempted to identify the target before they could provide their report in the presence of incentives. These designs may have artificially “encouraged” all participants to engage with the task. In this study, by contrast, we do not distinguish between disambiguation and report stages. Instead, we simultaneously reveal the target box to the participants and ask them to report the distance from their chosen box to the target. Financial incentives are also present on screen, as they were before, during the report. Importantly, given that there is no longer a distinct disambiguation stage, in this study design, participants are no longer forced to click on the target box. This design could potentially help the most dishonest participant to skip disambiguating the task, maintaining ambiguity, and directly provide a self-benefiting report. Therefore, the study tests the effectiveness of the training in a more conservative setting, when participants are not forced to engage with the task and therefore the effect of increasing their competence through ability training may not translate to decreased dishonesty. This study’s design and hypotheses were preregistered (https://osf.io/x4jd3/?view_only=61d2e89fcc4a4474b52743bdab488237).

### Method and Procedure

Methods and procedures remained largely identical to Studies 2a and 2b, with the main difference being the disambiguation and reporting stages condensed into one stage. This meant that after the target was revealed, participants were not required to click on the target. Instead, they were presented with an incentive table simultaneously with the target and were asked to report the distance to the target (for details about the methods see Appendix D).

### Results

**Figure 5.**
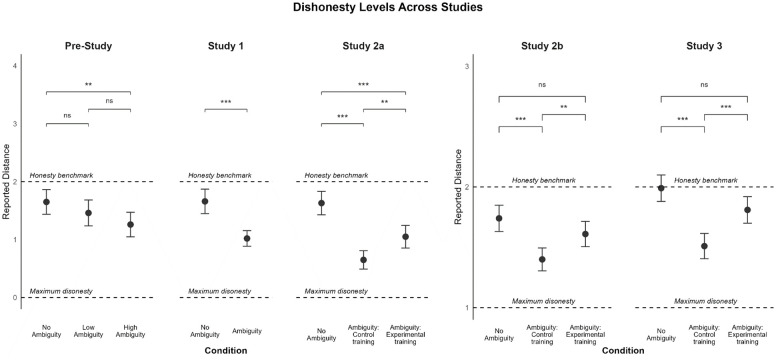
The Effect of Ambiguity and Training Manipulations on Dishonesty Levels Across Studies. *Note.* The No ambiguity condition always acts as the control condition. The Ambiguity conditions act as treatment conditions where higher dishonesty is expected in the Control Training conditions compared with the No Ambiguity and Experimental Training conditions.

To test for differences in dishonesty between the experimental conditions, we conducted a one-way between-subjects ANOVA. The results revealed a statistically significant difference in the reported distance between the conditions, *F*(2,586) = 20.83, *p* < .001. Pairwise comparisons showed significant differences between conditions, with the control condition (no ambiguity) associated with larger reported distances (*M* = 1.99, *SD* = 0.77) compared with the ambiguity condition with control training (*M* = 1.50, *SD* = 0.72, *p* < .001, Cohen’s *d* = 0.65). Thus, we replicated the main effect of ambiguity. Furthermore, the average report in the ability training condition was significantly higher than in the control training condition (*M* = 1.82, *SD* = 0.77, *p* < .001, Cohen’s *d* = 0.42) and slightly lower than in the no ambiguity condition (*p* = .071, Cohen’s *d* = 0.22). These results indicate that the training reduced dishonesty in situations where participants could effectively skip the disambiguation stage, supporting our main hypothesis. An illustration of the main effect in this study, as well as all previous studies, can be seen in Figure 5.

### Discussion

Taken together, these findings show that dishonesty can be lowered by rendering a task less ambiguous; however, one can also decrease dishonesty by targeting the individual rather than the task. In particular, our results suggest that when people become more capable of disambiguating information in the task they act less dishonestly. This emphasizes that dishonesty can be reduced by raising people’s ability levels, and providing them with the necessary skills to tackle ambiguity present in a task. We therefore highlight a novel approach to reduce dishonesty based on helping people to effectively resolve ambiguity in tasks.

Our results confirm our hypotheses: ambiguity leads to dishonesty (H1), disambiguation success leads to less dishonesty (H2), and disambiguation success can be fostered through brief ability training. These findings are summarized in our suggested model (Figure 1). We extend the literature on the relationship between ambiguity (i.e., ambiguous tasks) and dishonest behavior in several meaningful ways.

#### Disambiguation Success and Dishonesty

Our findings reveal that disambiguation success—that is, the successful resolution of ambiguity in a task—plays a critical role in reducing dishonest behavior. This means that those individuals who manage to make sense of ambiguous information, go on to act less dishonestly. We therefore show that benefits can be obtained when people successfully disambiguate a task. This had been hinted at previously by other researchers, in particular by Pascual-Ezama et al. (2020) who believed that discovering the truth makes an individual more bound to that truth. We show that when people successfully disambiguate a task, that is, discover the truth, this helps them to act more honesty. Researchers have shown that those who disengage from a task tend to act the most immorally (Bandura, 1991, 1997; Black et al., 2021; Pascual-Ezama et al., 2020). Advancing this line of thinking, we unraveled the link between disambiguation success and dishonesty, showing that those who solve ambiguity are more likely to behave honestly.

This suggests that resolving ambiguity reduces the moral justifications that are readily available in ambiguous conditions (Pittarello et al., 2015; Shalvi et al., 2012). Ambiguity, by nature, provides a “grey area” or “fog” that allows individuals to rationalize dishonesty without compromising their self-image, allowing them to maintain a moral self-concept while still acting in their own favor. This aligns with theories suggesting that failure to resolve ambiguity grants freedom to interpret information in self-serving ways (i.e., “moral wiggle-room,” Dana et al., 2007). Our findings demonstrate, for the first time, that successful disambiguation of a task can independently promote honesty, a pathway that extends beyond traditional interventions aimed at moral or ethical reminders.

#### Ability Training and Disambiguation Success

It is worth considering carefully the role that ability training can have in increasing people’s chances of successfully resolving an ambiguous task, leading to more truthful behavior. Previous research in this field has highlighted effective ways dishonesty can be reduced by targeting the individual, particularly by finding ways to stimulate an individual’s honest side (Loe & Weeks, 2000; Reynolds & Ceranic, 2007; Spoelma, 2021; Welsh & Ordóñez, 2014). The research most similar to ours is that of Black et al. (2021) who employed a training session to increase an individual’s sense of ethics, finding that the training increases the intention to act morally. The current research goes beyond moral primes and ethical training unraveling an effect that, on the surface, may appear far more surprising: that increasing a person’s ability levels decreases their tendency to act dishonestly in ambiguous tasks. Enhanced ability achieves this by increasing the odds a person will successfully disambiguate the task, helping them to better see through the fog of ambiguity. This is important because briefly training people to raise their ability levels, before an ambiguous task, is less likely to be interpreted as an attempt to steer behavior in a moral direction, in contrast to using moral primes or moral training, which people may experience as a limit to their personal freedom and cause the intervention to backfire (e.g., Berman & Johnson, 2015).

It is also interesting to appreciate the effect of ability enhancement on dishonesty by considering that moral primes are largely ineffective in conditions of unobservability, where participant behavior is not being individually monitored (Zhao et al., 2021). Because it is not possible, or even ethical, for organizations to constantly monitor employee behavior, devising interventions intended to be effective in conditions of unobservability, which was at the heart of the current investigation, is certainly valuable. In addition, measuring dishonesty in conditions of unobservability offers a glimpse into behavior that is unbridled by the presence of others. For practitioners, a further benefit of ability-based interventions as shown in this article, compared with moral primes and ethical training, is that enhanced ability levels can make it easier to cope with aversive situations and emotions (Bandura, 1977) that may be caused by ambiguous tasks.

#### Ambiguity Without Time Constraints

The link between ambiguity and dishonesty had been previously established in laboratory settings using tight time constraints (Leib et al., 2019; Lois & Wessa, 2021; Pittarello et al., 2015, 2019; Schweitzer & Hsee, 2002). However, when people are faced with ambiguous tasks in their everyday lives, such as conducting academic research or interpreting a document in a foreign language, they are usually free to exert as much effort as they want to solve the task. We show that when people are given as much time as they require then high levels of dishonesty emerge when the task is highly ambiguous. It has been previously suggested that time constraints bias decision-making toward reproachable behavior (Kruglanski & Freund, 1983; Shalvi et al., 2012), but here we see that the link between ambiguity and dishonest behavior continues to exist in the absence of time constraints.

#### Future Research

The current research examined dishonesty under conditions of unobservability, allowing participants to act as dishonestly as they wanted. While this approach captures unconstrained dishonesty, it measures dishonesty at the aggregate level, per condition, rather than at the individual level. Future research could benefit from identifying profiles most likely to exploit ambiguity or benefit from forms of training that increase their ability to disambiguate. Pascual-Ezama et al.’s (2020) work on dishonesty profiles provides a useful framework, although it did not consider ambiguity or disambiguation success. In addition, exploring variability in individuals’ ability to disambiguate, perhaps linked to personality traits, and the characteristics of those excelling in resolving ambiguity would be valuable.

In line with this, the relationship between disambiguation ability and honesty may not be linear. While those who are competent at resolving ambiguity in a particular task may act more honestly, as we show, it remains to be seen whether renowned experts who can see through the ambiguity better than most others in a given field (i.e., high finance) tend to behave most honestly. Extending this logic, research might examine traits that predict an individual’s likelihood of engaging in a disambiguation phase, as opposed to skipping an effortful interpretation altogether. This question is particularly relevant given that disambiguation appears crucial for promoting honesty. When considering diverse contexts, it may be fruitful to investigate whether the current results hold when misreporting reality for personal gain involves inward dishonesty, such as tracking diet progress or self-assessing performance, versus outward dishonesty directed at others, as in filing tax returns or loan applications.

Regarding ability training, it is noteworthy that ability, power, and dominance, while often linked (Anderson & Kilduff, 2009; Mast, 2010), are clearly distinct in their effects on dishonesty. While prior studies reveal links between power and dishonesty (Lammers et al., 2015) and between trait dominance and dishonesty (Kim & Guinote, 2022), they did not explicitly examine ambiguity. While these studies involving power did not explicitly examine ambiguity, the results appear to be in contrast with the current findings, whereby increased ability to disambiguate leads to less dishonesty. The divergence in effects between ability, power, and dominance underscores their distinctiveness and potentially opposing impacts in the realm of dishonesty. Only additional research will clarify whether ability levels and power lead to opposite predictions across all fields of dishonesty, whether points of convergence exist when tasks are ambiguous or not, or when the enhanced ability is accompanied by feelings of power.

Finally, this study investigated objective ability; the actual increase in one’s capacity to disambiguate information. It is unclear if these findings would hold if perceived ability, rather than objective ability, were increased. This might also be an interesting avenue for future research, especially given that feelings of entitlement can lead to dishonest behavior (Poon et al., 2013), suggesting that feelings of increased personal ability that are not reflected in reality may also cause the current results to flip.

#### Limitations

While ability training systematically reduced dishonesty in our studies by increasing people’s ability to disambiguate information in the task, it is worth considering alternative mechanisms that may not be entirely distinct. For example, by increasing people’s ability in the task through training, we might also have enhanced their attention to the task, making them more aware of the ethical considerations linked to their choices (Reynolds, 2006). Similarly, our training intervention may have inadvertently heightened participants’ sensitivity to self-incrimination. For example, the ability training may have prompted participants to feel more involved in the task or even triggered a sense of reciprocity or gratitude toward the experimenters. This increased involvement or sense of reciprocity, combined with a clearer understanding of the task, might have made participants more cautious about appearing dishonest, particularly by avoiding extremely dishonest responses that could raise internal concerns of self-incrimination. Although we found no direct evidence for this effect, it is worth considering when interpreting our results. Future studies could investigate these alternative explanations to disentangle the effects of disambiguation success from other motivational factors potentially triggered by the ability training procedure.

## Conclusion

Our results suggest that public, private, and non-profit organizations can reduce dishonesty by either reducing ambiguity present in tasks or by training individuals to better tackle ambiguity. A typical and frequent real-life example includes training employees on how to comply with a given procedure. All forms of brief and cost-effective training that help people disambiguate a given setting, or clarify the procedures within such a setting, fall within the class of interventions we propose here. In conclusion, we decided to place our scientific lens on the individual experiencing ambiguity rather than solely on the ambiguity in the task or environment. Once that shift of focus was made, the importance of investigating one’s ability to successfully disambiguate a task became immediately apparent. If less ambiguity leads to less dishonesty, as the literature amply suggests, then individuals who no longer perceive ambiguity—because they have successfully disambiguated the task—should behave less dishonestly. That is what we found in all our studies. The removal of ambiguity from tasks appears to effectively silence those justifications and excuses for acting dishonestly, that are so readily available to people when ambiguity is present. We, therefore, show that dishonesty can be lowered by removing ambiguity from the environment, making tasks clearer and more immediately understandable, or equally by targeting the individual, helping them to see through the fog of ambiguity.

## Supplemental Material

sj-pdf-1-psp-10.1177_01461672241305687 – Supplemental material for Seeing Through the Fog: The Ability to Resolve Ambiguity Reduces Dishonesty Supplemental material, sj-pdf-1-psp-10.1177_01461672241305687 for Seeing Through the Fog: The Ability to Resolve Ambiguity Reduces Dishonesty by Michael Puntiroli, Serhiy Kandul, Valéry Bezençon and Bruno Lanz in Personality and Social Psychology Bulletin
