# Supplementary material for: Seeing Through the Fog: The Ability to Resolve Ambiguity Reduces Dishonesty
Source: Pers Soc Psychol Bull. 2025 Jan 7;52(5):1299–313. doi: 10.1177/01461672241305687 (PMC13022029; doi:10.1177/01461672241305687)
Supplement: sj-pdf-1-psp-10.1177_01461672241305687 – Supplemental material for Seeing Through the Fog: The Ability to Resolve Ambiguity Reduces Dishonesty [file sj-pdf-1-psp-10.1177_01461672241305687.pdf]

Appendix A

Pre-Study: Validation of Method and Replication of Previous Findings

Participants. Our first prediction is that increased ambiguity present in the task leads to more dishonesty. Using the statistical program G*Power 3.1 (Faul et al., 2009), we estimated that a minimum sample size of 390 was necessary given our predicted medium-sized effect (f2 = 0.20), given $\alpha = 0.05$, using three independent groups. We proceeded to recruit 450 participants; however, three did not complete the study. Our final sample consists of 447 participants ($M_{Age} = 36$; 38% males).

Materials and Procedures. Employing a between-subjects design, participants were required to identify the target bead under No, Low, or High ambiguity settings. Once participants identified the target bead, they were asked to report the distance from their guessed bead to the target bead. The distance was counted clockwise (Figure 1-S). Before entering their reported distance, participants viewed an incentive scheme instructing them that the closer they were to the target the higher their bonus payment would be.

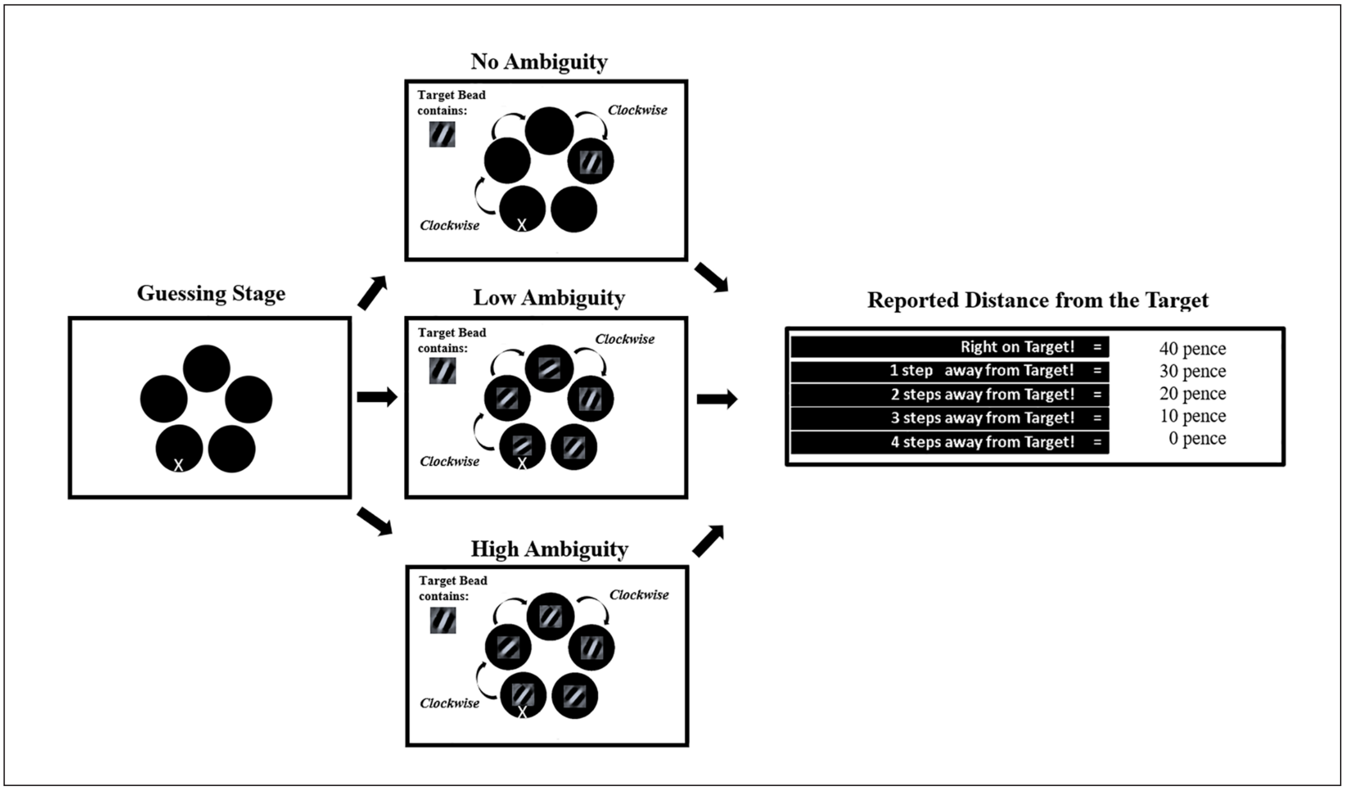

Figure 1-S. The Experimental Sequence Presented to Participants Is Displayed.
Note. Participants were first asked to guess the location of the target bead and to remember their choice (Guessing Stage). In this example, the participant guessed the bottom left bead, indicated with a white cross. Then participants identified the target bead in one of three possible ambiguity conditions, No, Low, or High Ambiguity, depending on the presence or not of distractor patches, and how similar the distractor patches were to the patch presented on the target bead. The participant then counted the distance from their chosen bead to the target bead. In this example, the target bead was to the right, in the 3 o'clock position in all conditions, and was therefore three clockwise steps away from the chosen bead. Finally, participants reported how close they were to the target bead (i.e., the distance).

Results. To detect mean-based differences between the three conditions, we ran a one-way between-subjects ANOVA, expecting that higher ambiguity would lead to more dishonesty. We found a significant difference in "reported distance" between the conditions, $F(2, 446) = 3.82$, $p < .023$, with planned comparisons revealing that participants reported being significantly closer to the target in the high ambiguity condition ($M = 1.23$; $SD = 1.28$) compared with the no ambiguity condition (control) ($M = 1.65$; $SD = 1.34$; mean difference = 0.42, $SE = 0.15$, $p = .006$; CI: [.12, .72], Cohen's d=0.32). No differences emerged between No Ambiguity and Low Ambiguity ($p = .231$, CI: [−.12, .49]). The results are summarized in Figure 2-S.

These results showed that people acted more dishonestly the more their task was ambiguous, thus confirming previous results and H1. Sample size will be increased in the following experiments to be able to detect smaller effect sizes.

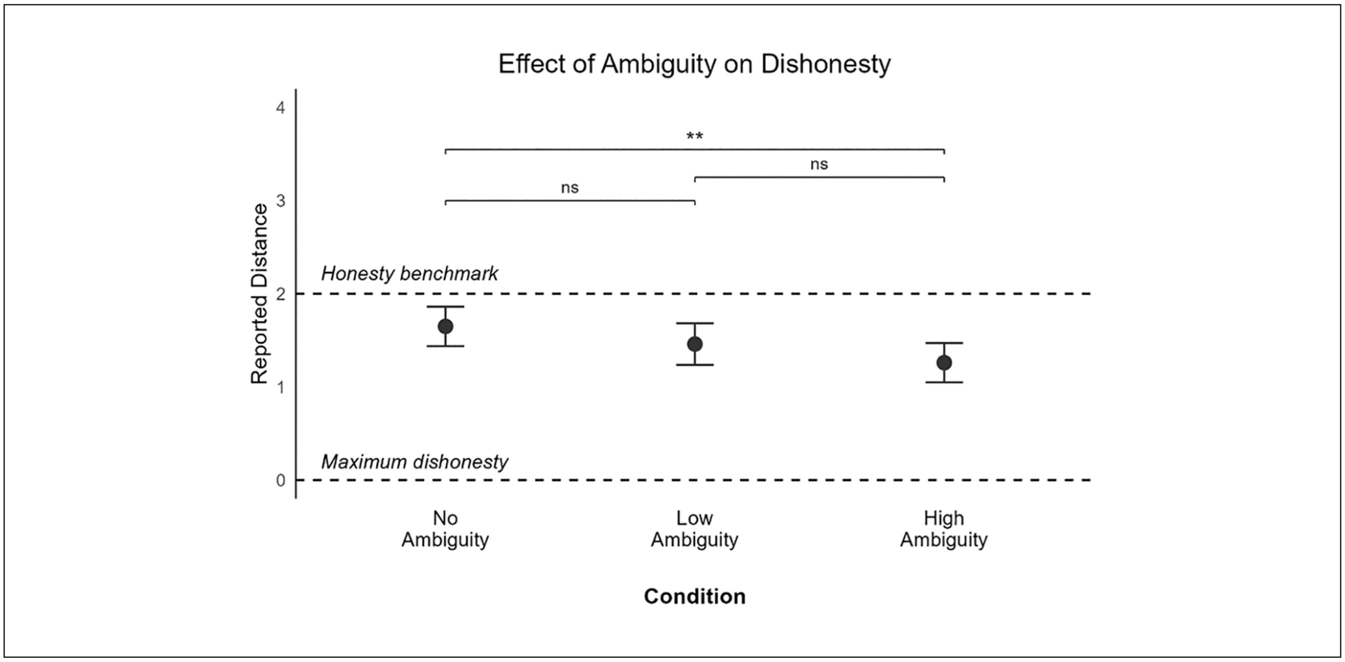

Figure 2-S. Illustration of the Distance From the Target Reported by Participants in Each of the Ambiguity Conditions.
Note. The closer participants' chosen bead was to the target, the more money they were paid. Shorter distances to the target signify more dishonesty. The dashed line on y-axis represents a theoretical benchmark of complete honesty. Error bars represent the standard error of the mean (±2).
*p <.05. **p<.01.

Appendix B

Study 1 Mediation Analysis

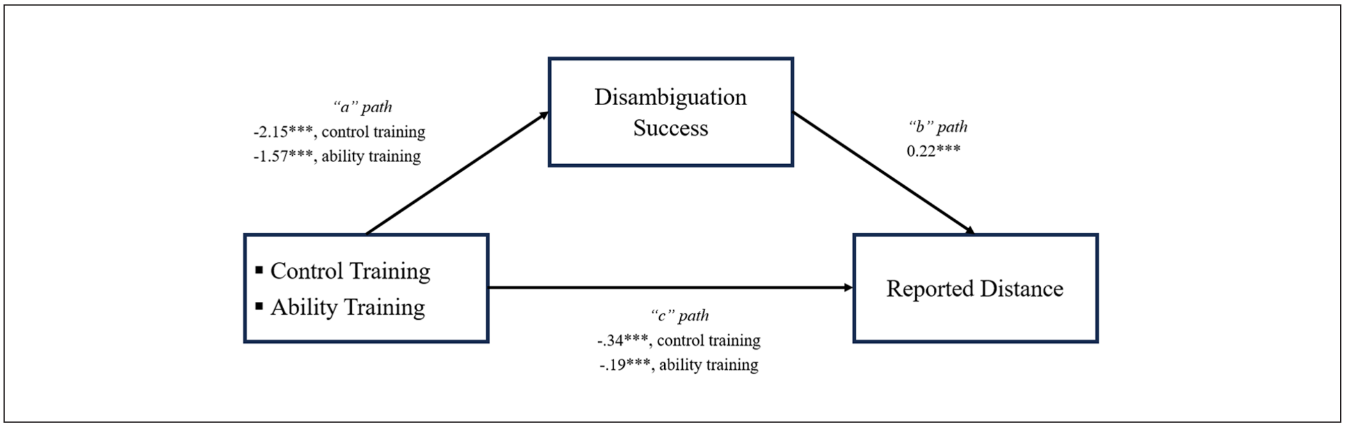

Figure 3-S. Effect of Training Partially Mediated by the Ability to Solve the Task.
Note. Reference category= control condition with no ambiguity. Each training condition presents ambiguity and is compared with a control condition with no ambiguity. Significant negative coefficients when comparing training conditions to the control indicate the size of the advantage of the control condition.
*p<.05. **p<.01. ***p<.001.

Appendix C

Study 1 Insights

By looking separately at the quantiles based on time spent on the task, we aim to glean additional insights into the interplay between disambiguation success, effort, and dishonesty. We will focus on the behavioral profiles identified by Pascual-Ezama et al. (2020), which are those of truthtellers, liars, and cheaters (see profiles described in introduction).

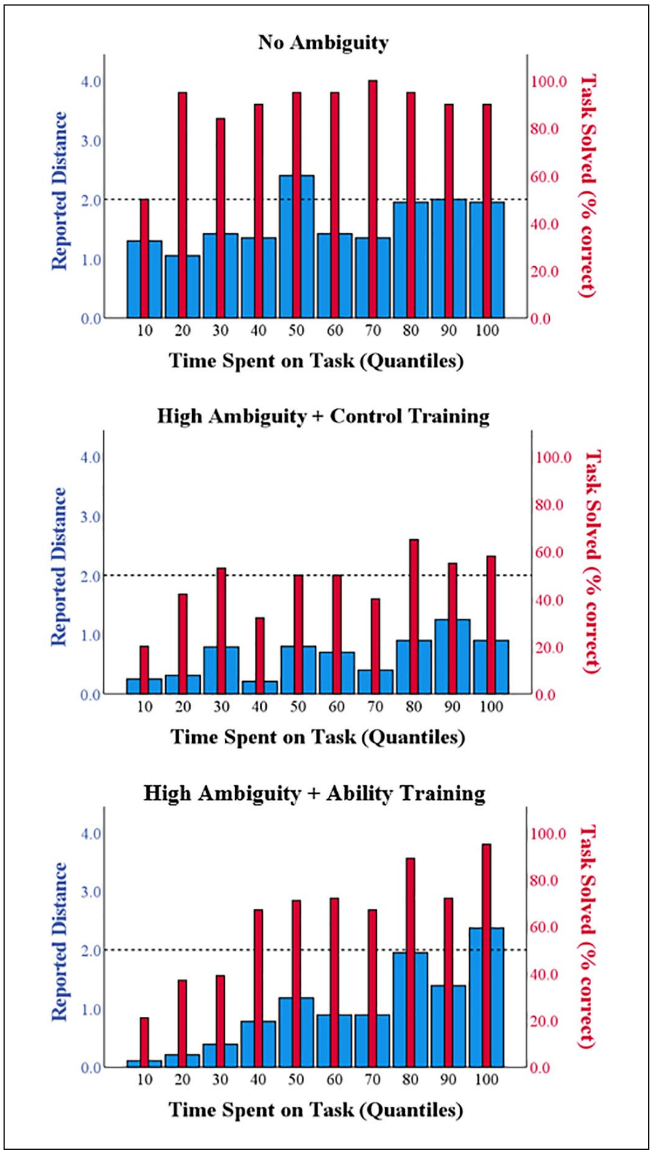

Figure 4-S. Illustration of the "Reported Distance" and "Disambiguation Success" Per Quantile, Indicating the "Time Spent on the Task" Disambiguating the Target From the Distractors, that is "Effort".

Note. Shorter distances to the target signify more dishonesty. The dashed line on y-axis represents a theoretical benchmark of complete honesty. Higher levels of disambiguation success and reported distance (i.e., less dishonesty) can be seen in the ability training condition compared with the control training condition. Each bar represents N=20 (+-1).

The first quartiles can be particularly telling, relating to people who spent little to no effort on the task. We, therefore, decided to initially focus only on the first three quantiles (10%, 20%, and 30%, that is, the 30% who spent the least amount of time on the task) and run a MANOVA with the conditions as an independent variable and reported distance and disambiguation success as dependent variables. We observed a main effect on reported distance, $F(2, 173) = 15.43$; $p < .001$; partial $\eta^2 = .15$, and disambiguation success, $F(2, 173) = 15.56$; $p < .001$; partial $\eta^2 = .16$. Planned comparisons highlighted no differences between control training and ability training on reported distance (S.E. = .20, p =.271) or on task solved (S.E. = .09, p =.506), while the no ambiguity condition differed significantly from the two training conditions across both dimensions (all p <.001). Therefore, the ability training has no beneficial effect on people in these first quartiles, compared to the control training (Figure 4-S). The cheaters who tend to put no effort into solving the task, appear to remain unperturbed by the intervention.

Next, we decided to focus only on truthtellers and liars, who are those who certainly attempt to solve the task, then either act truthfully (i.e., truthtellers) or not (i.e., liars). We therefore selected cases in the last three quantiles (80%, 90% and 100%) and ran a MANOVA once again, which highlighted a main effect on reported distance, $F(2, 171) = 5.58$; $p < .001$; partial η2 = .09, and disambiguation success, $F(2, 171) = 11.53$; $p < .001$; partial $\eta^2 = .12$. This time the planned comparisons highlighted significant differences between control training and ability training conditions on reported distance (SE = .26, p <.001) and on task solved (SE = .07, p <.001). Also interestingly, this time no differences emerged between control and ability training conditions on reported distance (SE = .26, p =.831) or on disambiguation success (SE = .07, p =.423). Finally, the control and control training conditions differed significantly across reported distance and disambiguation success (both p <.001). This implied that the beneficial effect of ability training is mainly observed in these higher quartiles, therefore helping the liars become truthtellers.

Appendix D

Study 3. Method and Participant Details

As in Studies 2a and 2b, we employed a between-subjects design where we manipulated the level of ambiguity and the presence of training to resolve ambiguity in the task. Participants were randomly assigned to one of three conditions: a "control" (no ambiguity) condition, a "control training" condition, and an "ability training" condition. After providing them with a basic description of the study, we obtained their consent to participate. The control training condition aimed to familiarize participants with the task without improving their ability to disambiguate it. In contrast, the ability training condition was designed to enhance participants' ability to disambiguate the task. We aimed to

recruit 600 participants but ultimately interrupted data collection once we achieved 594 participants from prolific.co.uk (*M*Age = 38; 49% male), as data collection was slowing down considerably and we did not want this process to extend past sunset, when participants may be less alert. After excluding 5 participants who failed a simple attention check, our final sample consisted of 589 participants.

Study 3. Materials and Procedure Details

We largely retained the procedure from Studies 2a and 2b, including identical stimuli and incentives for (mis)reporting. The main differences involved the disambiguation stage. In this study, after the target was revealed, participants were not required to click on the target, that is, no forced disambiguation. Participants were instead presented with an incentive table simultaneously with the target and were asked to report the distance from their chosen box to the target. In addition, we removed explicit references to disambiguation in the instructions, such as by asking participants to report the distance without mentioning the need to count it first. Thus, participants were completely free to decide how much effort they wanted to put into identifying the target (they could, in fact, skip the disambiguation altogether and report immediately).

All participants were initially presented with three examples clarifying the procedure. In the control "no ambiguity" condition ($N = 199$), participants underwent no training and proceeded straight to the main task. In the "control training" condition ($N = 192$), participants completed five practice rounds in which they identified the target box, before proceeding to the main task. These practice rounds contained no ambiguity; the target box was surrounded by boxes with only one black dot inside them, as in the control condition. In the "ability training" condition ($N = 197$), participants also completed five practice rounds, but they had to identify the target box containing three black dots among non-target boxes also containing three dots. As in Study 2b, these distracting non-target boxes contained two black dots and one dark gray dot that could be mistaken for black. Participants continued attempting to select the target box until they selected the correct one.
